# Foxtail millet (*Setaria italica* (L.) P. Beauv) CIPKs are responsive to ABA and abiotic stresses

**DOI:** 10.1371/journal.pone.0225091

**Published:** 2019-11-12

**Authors:** Jinfeng Zhao, Aili Yu, Yanwei Du, Gaohong Wang, Yanfang Li, Genyou Zhao, Xiangdong Wang, Wenzhong Zhang, Kai Cheng, Xin Liu, Zhenhua Wang, Yuwen Wang

**Affiliations:** 1 Millet Research Institute, Shanxi Academy of Agricultural Sciences, Shanxi Key Laboratory of Genetic Resources and Breeding in Minor Crops, Changzhi, Shanxi, People's Republic of China; 2 Tangshan Academy of Agricultural Sciences, Tangshan, Hebei, People's Republic of China; National Institute of Plant Genome Research, INDIA

## Abstract

CBL-interacting protein kinases (CIPKs) have been shown to regulate a variety of environmental stress-related signalling pathways in plants. Foxtail millet (*Setaria italica* (L.) P. Beauv) is known worldwide as a relatively stress-tolerant C_4_ crop species. Although the foxtail millet genome sequence has been released, little is known about the functions of CIPKs in foxtail millet. Therefore, a systematic genome-wide analysis of CIPK genes in foxtail millet was performed. In total, 35 CIPK members were identified in foxtail millet and divided into four subgroups (I to IV) on the basis of their phylogenetic relationships. Phylogenetic and gene structure analyses clearly divided all SiCIPKs into intron-poor and intron-rich clades. Cis-element analysis subsequently indicated that these SiCIPKs may be involved in responses to abiotic stimuli, hormones, and light signalling during plant growth and development, and stress-induced expression profile analysis revealed that all the SiCIPKs are involved in various stress signalling pathways. These results suggest that the CIPK genes in foxtail millet exhibit the basic characteristics of CIPK family members and play important roles in response to abiotic stresses. The results of this study will contribute to future functional characterization of abiotic stress responses mediated by CIPKs in foxtail millet.

## Introduction

It is well known that abiotic stresses such as drought, salinity and low temperature severely affect the yield and quality of crops. To cope with such adverse conditions, plants have developed elaborate and systematic mechanisms during their evolution [1]. Many CBL-interacting protein kinases (CIPKs) are involved in the response and adaptation to stresses [2]. The genes encoding these serine/threonine protein kinases constitute an important, widespread multigene family in the plant kingdom [3]. Sequence analysis has indicated that CIPK proteins have a relatively conserved N-terminal kinase domain and a less conserved C-terminal regulatory domain [4]. A highly conserved 24-amino acid region within the C-terminal regulatory domain of CIPK designated as the NAF domain (Pfam No. PF03822) is required for interaction with CBLs during exposure to stress [5].

CIPKs have been found to play a very important role in response to external stress stimuli. The first CIPK identified in plants was shown to be involved in the *Arabidopsis* salt overly sensitive (SOS) pathway [6]. AtCBL4 (AtSOS3) can interact with AtCIPK24 (AtSOS2), and this complex specifically regulates signals in response to salt stress [7, 8]. Moreover, the function of genes homologous to *AtCIPK24* in other species, such as *MdCIPK6L*, *MdSOS2*, *ZmCIPK16*, and *SiCIPK24*, is similar to that of *AtCIPK24* (*AtSOS2*), which involves increasing plant tolerance to salt stress [9–12]. In addition, AtCIPK23 reportedly forms a complex with AtCBL1 and AtCBL9 to regulate potassium homeostasis under low-potassium stress [13], and *AtCIPK7* may play a role in the response to cold by interacting with AtCBL1 [14]. Furthermore, by increasing the expression levels of drought-related genes, *OsCIPK23* can improve the drought tolerance of rice when this gene is overexpressed [15], and transgenic cotton plants overexpressing *GhCIPK6* were shown to exhibit increased tolerance to many abiotic stresses [16]. Moreover, some CIPKs from different plant species have been characterized as participating in plant development and hormone signalling [17, 18]. For example, *GhCBL*/*CIPK* genes were recently reported to play a critical role in the regulation of cotton fibre elongation [19], and *CaCIPK6* (from *Cicer arietinum*) is involved in auxin transport and root development as well as response to salt stresses [20]. *AtCIPK8* is involved in early nitrate signalling and regulates the low-affinity phase of the primary nitrate response [21]. In contrast, *AtCIPK1* is involved in stress-response pathways and represents a cross-talk node that integrates abscisic acid (ABA)-dependent and ABA-independent aspects of abiotic stress-related signalling [22]. Some CBL/CIPK genes in major crop species such as rice, wheat, sorghum and cassava have also been identified and functionally analysed [23–27]. Recent research has further revealed that the CBL/CIPK network in plants plays an important role in responses to abiotic stresses. The expression of wheat *TaCIPK23* is induced by multiple abiotic stresses and interacts with TaCBL1 on the plasma membrane. Overexpression of *TaCIPK23* confers a higher survival rate to transgenic plants than to nontransgenic plants under drought conditions [28]. The BrCIPK1 protein interacts with OsCBL1 and OsCBL5. Furthermore, compared with nontransgenic rice lines, *BrCIPK1* transgenic rice lines present significantly higher biomass, water content, and proline and free sugar contents [29]. Bimolecular fluorescence complementation (BiFC) and yeast two-hybrid experiments revealed that ZmCIPK8 interacts with ZmCBL1, ZmCBL4 and ZmCBL9. Moreover, qRT-PCR analysis revealed that *ZmCIPK8* is strongly induced by drought stress in maize leaves and roots, and overexpression of *ZmCIPK8* in tobacco increased the drought tolerance of transgenic tobacco seedlings and induced the expression of the NAC, CBF, and Rd29A genes [30]. Twelve *HsCIPK* genes were isolated from annual wild barley growing on the Tibetan Plateau; further research suggested that these *HsCIPK*s are involved in responses to heavy metal toxicities and other abiotic stresses [31]. In cassava, MeCIPK23 was shown to interact with MeCBL1 and MeCBL9, and overexpression of these genes conferred an improved defence response to transgenic plants. Virus-induced gene silencing of *MeCIPK23* or *MeCBL1/9* or both genes resulted in disease sensitivity [32]. In another study, *PsCIPK* was cloned from *Prunus serrulata* ‘Yimeng’ and analysed in both submergence-tolerant and submergence-sensitive accessions under submergence stress. The results showed that *PsCIPK* influenced the expression of genes related to carbohydrate metabolism and plant growth (PsPDC, PsSUS, PsRAMY, and PsEXP) to different extents under submergence stress and during recovery, systematically improving submergence resistance [33]. *BnCIPK9* is strongly induced by wounding stress, and overexpression of *BnCIPK9* reduces oil synthesis in transgenic *Brassica napus* plants during seed development. Functional analysis suggested that *CIPK9*, *CBL2*, and *CBL3* might work together and play similar roles in root establishment under sugar-free conditions [34]. In summary, the above research indicates that CIPKs have various physiological functions in response to multiple stress stimuli, especially abiotic stresses.

Foxtail millet (*Setaria italica* (L.) Beauv) is a C_4_ graminaceous crop species that originates from China, has a long (approximately 7,000 years) history of cultivation and is widely planted in northern China and in other Asian countries [35]. Foxtail millet is a hardy cereal known for its superior tolerance to drought, salinity, and diseases; high nitrogen- and water-use efficiencies; and nutritional properties [36]. The potential abiotic stress tolerance of foxtail millet has motivated the research community to study the molecular mechanisms of this species [37]. Although the foxtail millet genome sequence has been completed and released [35, 38], the CIPK gene family in foxtail millet has not been fully characterized. Therefore, it is very important to identify CIPK genes in foxtail millet and evaluate their different responses to abiotic stresses. In this study, we identified 35 CIPK genes from the foxtail millet genome and analysed their genetic organization, gene structure and conserved motifs, evolution, and stress-related cis-elements. We also evaluated the gene expression profiles of the SiCIPKs under ABA and abiotic stresses. These systematic analyses revealed that the CIPKs in foxtail millet exhibit the basic characteristics of CIPK family members and may be important contributors to the superior tolerance of this species. The SiCIPK genes reported in this study will enrich our knowledge of CIPKs in the plant kingdom, and our results lay a foundation for revealing the functions and mechanisms of stress responses mediated by the CBL/CIPK pathway in foxtail millet.

## Materials and methods

### Genome-wide identification of CIPK genes in foxtail millet

To identify the genes encoding CIPK proteins in foxtail millet, the 8.3X assembled version (V2.2) of the foxtail millet genome was downloaded from Phytozome V12.1 (https://genome.jgi.doe.gov) [38]. The CIPK genes were identified via the methods described by Hu [3]. Each CIPK candidate sequence was examined for the presence of the NAF domain and protein kinase domain necessary for consideration as a member of the CIPK family.

### Protein properties and sequence analyses

The protein parameters were evaluated, and sequence analysis was performed according to the method described by Tang [39]. Prediction of the molecular weight (MW) and isoelectric point (pI) of each SiCIPK protein was conducted using the online tool compute pI/Mw (http://web.expasy.org/compute_pi/). Motif prediction of SiCIPKs was carried out using MEME software (http://meme-suite.org/tools/meme) [40]. The maximum number of domains was set to 15, the width of functional domains was set to 6–60, and the other parameters were set to the default values. The motifs identified by MEME software were annotated on the PROSITE website (http://prosite.expasy.org/). The Gene Structure Display Server (GSDS 2.0, http://gsds.cbi.pku.edu.cn/) was then used to analyse the SiCIPK gene structure by comparing cDNAs with the corresponding genomic sequences [41].

### Chromosomal locations and promoter analyses

Chromosome positional information for the SiCIPK genes was collected from the JGI database (https://phytozome.jgi.doe.gov/) [38], and images were drawn by MapChart 2.30 software. The sequences 1,500 bp upstream of the coding region of the SiCIPK genes were obtained from the JGI database (https://phytozome.jgi.doe.gov/) [38], and cis-regulatory elements were identified by PlantCARE (http://bioinformatics.psb.ugent.be/webtools/plantcare/html/) software [42].

### Multiple sequence alignment and phylogenetic analysis

The amino acid sequences of CIPKs from *Arabidopsis*, rice, maize, sorghum, *Picea*, moss, green algae, and fungi were retrieved from the JGI (https://phytozome.jgi.doe.gov/), MaizeGDB (https://www.maizegdb.org/) and NCBI (https://www.ncbi.nlm.nih.gov/) databases. ClustalW 2.0 software was subsequently used for multiple sequence alignment analysis [43]. Phylogenetic trees were constructed via the neighbour-joining (NJ) and maximum likelihood (ML) methods by MEGA 6.0 software [44].

### Plant materials, growth conditions and treatments

Seeds of the foxtail millet cultivar Yugu 1 were obtained from the Millet Research Institute, Shanxi Academy of Agricultural Sciences, sown in pots and grown in a controlled incubator (16-h light/8-h dark photoperiod, 28°C day/20°C night temperature, 60–70% humidity) [12]. After reaching the three-leaf stage, the seedlings were transferred and precultured for 24 h in half-strength Hoagland's solution with aeration. Stress treatments were applied by transferring the seedlings to the same solution containing 20% polyethylene glycol (PEG) (MW of 6,000), 250 mM NaCl or 100 μM (+)-cis, trans-ABA [12]. For cold treatment, the seedlings were transferred to a growth chamber set at 4°C. Whole plants under a time series treatment (0, 3, 6, 12 and 24 h; three biological replicates) were harvested, immediately frozen in liquid nitrogen and stored at -80°C for RNA extraction.

### Real-time quantitative PCR (RT-qPCR)

Total RNA was extracted from the samples via RNAiso reagent (TaKaRa, Dalian, China) according to the manufacturer’s instructions. Expression of the SiCIPK genes in response to PEG, salt, cold and ABA was examined by RT-qPCR analysis via SYBR Green qPCR Master Mix (TaKaRa, Dalian, China) in conjunction with a LightCycler 480 instrument (Roche Diagnostics, Penzberg, Germany). PCR was performed according to the manufacturer's protocol. Each 20-μL amplified PCR mixture comprised the following components: 10 μL of 2X SYBR Green qPCR Master Mix (BBI, Shanghai, China), 2 μL of cDNA template, 0.4 μL of each primer (10 μM), and 7.2 μL of sterile distilled water. The PCR procedure consisted of 95°C pre-denaturation for 3 min, followed by 45 cycles of 95°C denaturation for 7 s, 57°C annealing for 10 s and 72°C extension for 15 s. Gene-specific primers were designed with Primer Premier 5.0 software on the basis of the nonconserved sequence of each SiCIPK gene. The β-actin gene of foxtail millet (Seita.7G294000) was used as an internal control for normalization, as described by Xu [45]. All primers used are listed in S1 Table. RT-PCR was performed three times for each biological sample, and the relative expression of the SiCIPKs under the different stresses was calculated according to the relative 2^-ΔΔCt^ method [46]. The values are given as the means ± SEs of three different experiments with three replicate measurements. Statistical analysis was performed via Student's t test.

## Results

### The foxtail millet genome encodes 35 CIPK genes

We searched for candidate CIPK genes in the foxtail millet genome according to the methods described by Hu [3]. After the genes were systematically analysed, 35 SiCIPKs were identified as candidate CIPK genes. To facilitate recording, we named these genes according to their corresponding locations in the genome. Like other CIPKs, all identified SiCIPKs contain a conserved N-terminal catalytic kinase domain and a C-terminal regulatory domain (S1 Fig). In addition, all SiCIPKs possess an activation domain within the N-terminal sequence and a highly conserved NAF domain within the C-terminal sequence (S1 Fig). The proteins range in size between 402 and 983 amino acids. The relative MWs vary from 45.51 to 112.42 kD, and most (77.14%) have high pIs (pI > 7.0). Detailed information concerning various CIPK parameters is provided in Table 1.

**Table 1:** Features of CIPK genes in foxtail millet.

Name	Gene ID (Phytozome)	Chromosomal location	Gene length (bp)	Amino acid length (aa)	PI	MW (kD)	Intro	CDS length (bp)
*SiCIPK1*	Seita.1G065400	scaffold_1:6114480-.6119211	4732	442	5.82	49.52	13	1329
*SiCIPK2*	Seita.1G079400	scaffold_1:7157400–7160348	2949	463	9.06	53.14	1	1392
*SiCIPK3*	Seita.2G032200	scaffold_2:2622179–2626013	3835	449	9.28	50.70	12	1350
*SiCIPK4*	Seita.2G206200	scaffold_2:30757672–30759715	2044	473	8.81	51.37	1	1422
*SiCIPK5*	Seita.2G405500	scaffold_2:46639620–46644650	5031	444	8.56	49.91	12	1335
*SiCIPK6*	Seita.2G431900	scaffold_2:48228937–48232337	3401	432	7.68	46.74	1	1299
*SiCIPK7*	Seita.2G432000	scaffold_2:48253461–48256377	2917	450	9.28	51.09	1	1353
*SiCIPK8*	Seita.2G439100	scaffold_2:48819266–48823750	4485	447	7.53	50.59	14	1344
*SiCIPK9*	Seita.3G053800	scaffold_3:3408823–3413527	4705	472	6.55	52.40	11	1419
*SiCIPK10*	Seita.3G181100	scaffold_3:13673885–13675761	1877	402	5.52	45.51	0	1209
*SiCIPK11*	Seita.3G205700	scaffold_3:15929206–15930558	1353	416	9.60	47.02	2	1251
*SiCIPK12*	Seita.3G284500	scaffold_3:26688377–26689696	1320	439	8.98	48.40	0	1320
*SiCIPK13*	Seita.3G285000	scaffold_3:26831444–26836199	4756	459	9.03	51.87	1	1380
*SiCIPK14*	Seita.3G380200	scaffold_3:48267583–48269360	1778	436	9.23	47.53	0	1311
*SiCIPK15*	Seita.4G175800	scaffold_4:28216455–28217894	1440	479	8.78	52.66	0	1440
*SiCIPK16*	Seita.4G221700	scaffold_4:34148605–34155126	6522	452	8.52	50.84	13	1359
*SiCIPK17*	Seita.5G031200	scaffold_5:2983068–2989104	6037	463	6.58	52.02	12	1392
*SiCIPK18*	Seita.5G145700	scaffold_5:12942864–12944429	1566	521	6.73	57.45	0	1566
*SiCIPK19*	Seita.5G145900	scaffold_5:12948718–12950070	1353	450	9.23	51.12	0	1353
*SiCIPK20*	Seita.5G190200	scaffold_5:24367119–24374943	7825	450	6.35	50.63	14	1353
*SiCIPK21*	Seita.5G325400	scaffold_5:37488769–37490729	1961	476	9.22	53.08	1	1431
*SiCIPK22*	Seita.5G325500	scaffold_5:37492716–37496574	3859	518	8.92	57.25	1	1557
*SiCIPK23*	Seita.5G364100	scaffold_5:40228601–40231852	3252	508	8.73	57.56	1	1527
*SiCIPK24*	Seita.6G163200	scaffold_6:28556400–28558663	2264	451	9.14	48.83	0	1356
*SiCIPK25*	Seita.7G300300	scaffold_7:34092477–34095321	2845	444	9.10	50.76	1	1335
*SiCIPK26*	Seita.7G311200	scaffold_7:34704000–34709429	5430	440	8.05	50.46	14	1323
*SiCIPK27*	Seita.8G003400	scaffold_8:222430–227696	5267	440	7.66	50.35	14	1323
*SiCIPK28*	Seita.8G014400	scaffold_8:841196–844251	3056	445	9.11	50.92	1	1338
*SiCIPK29*	Seita.8G171900	scaffold_8:31465259–31475552	10294	981	5.85	111.88	20	2946
*SiCIPK30*	Seita.8G172100	scaffold_8:31487716–31498216	10501	983	5.91	112.42	20	2952
*SiCIPK31*	Seita.9G162500	scaffold_9:10895437–10897187	1751	431	9.03	46.88	0	1296
*SiCIPK32*	Seita.9G411700	scaffold_9:46963528–46966563	3036	449	9.22	51.00	1	1350
*SiCIPK33*	Seita.9G422900	scaffold_9:47895220–47900463	5244	449	8.25	50.74	14	1350
*SiCIPK34*	Seita.9G557900	scaffold_9:57360257–57364586	4330	452	7.99	50.83	13	1359
*SiCIPK35*	Seita.J025400	scaffold_47:9096–13084	3989	433	7.61	49.64	13	1302

Abbreviations:bp = base pair, aa = amino acids, PI = isoelectric point, MW = molecular weight, kD = kilodaltons

### Genomic location, phylogeny and genetic organization of the SiCIPK gene family

**Fig 1:**
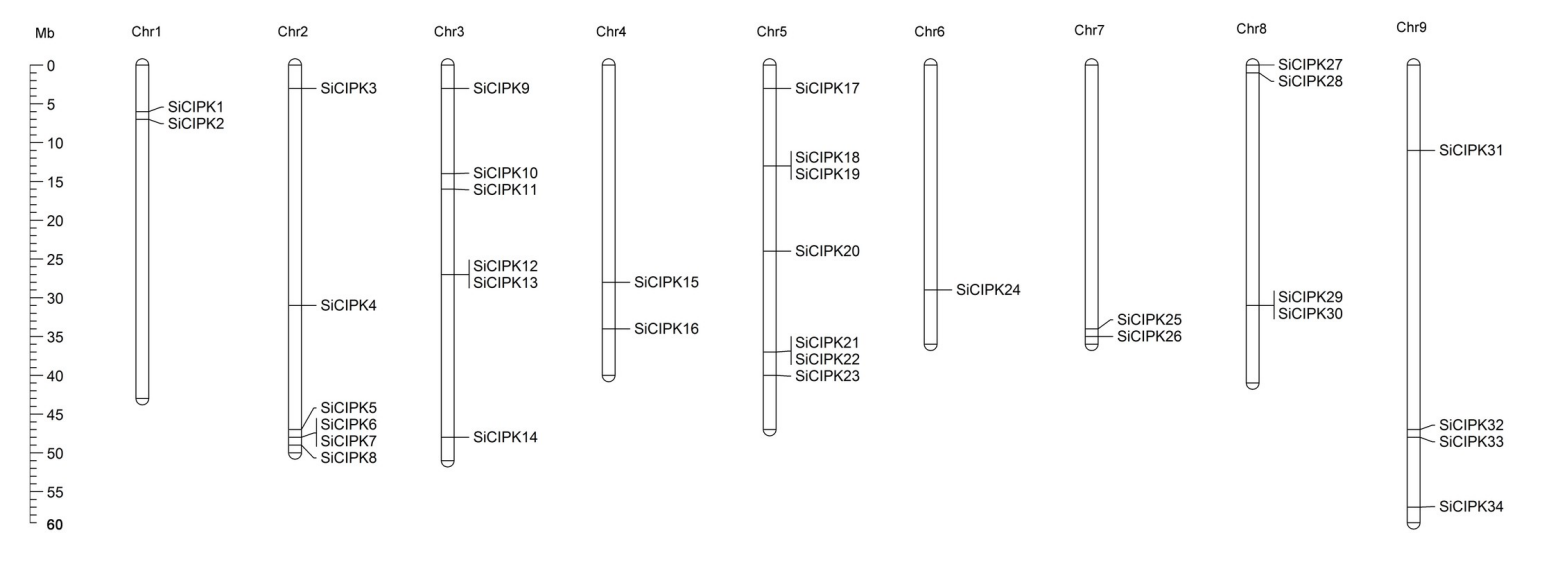
Distribution of identified SiCIPK genes among the nine foxtail millet chromosomes. The number in megabases (Mb) on the left represents the physical location of the corresponding SiCIPK gene. The Roman numbers above each chromosome represent the corresponding chromosome in foxtail millet. The vertical bars represent the chromosomal positions of the SiCIPK genes, and the gene names are on the right.

**Fig 2:**
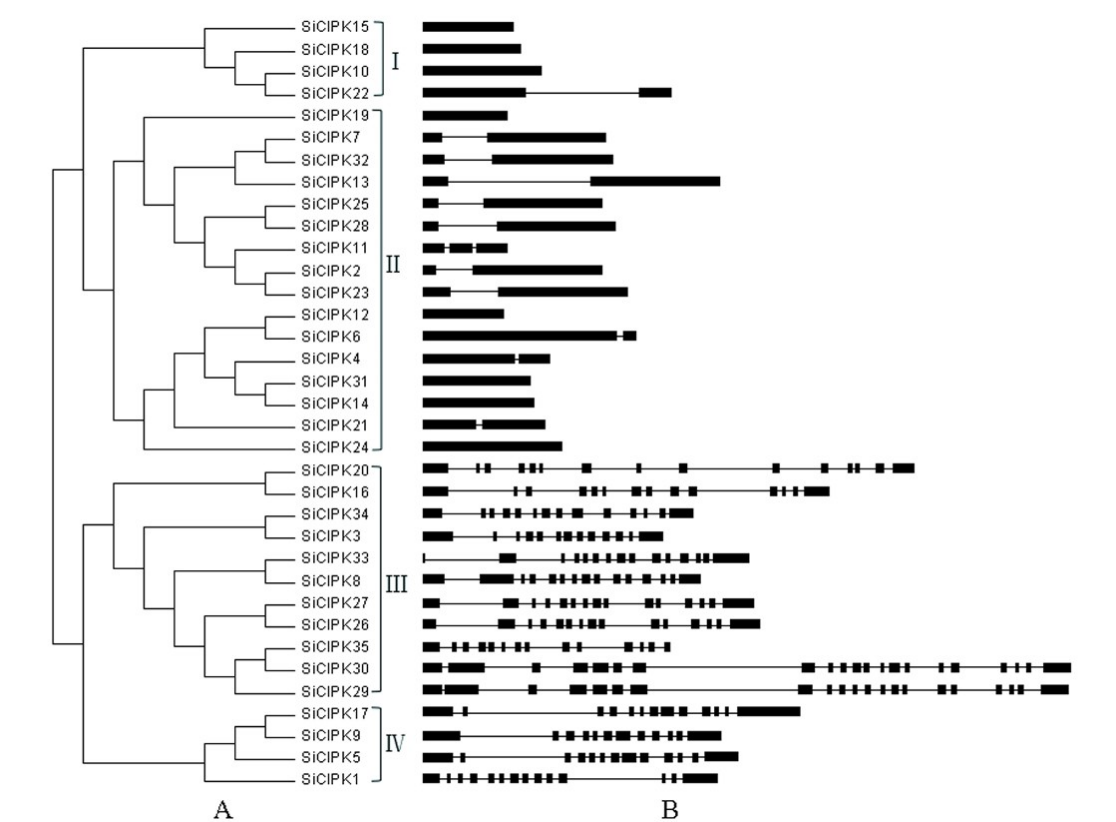
Phylogenetic relationships and exon-intron structures of the SiCIPK family. (A). Phylogenetic relationships of the CIPK family in foxtail millet. (B). Exon-intron structure analyses of the CIPK family in foxtail millet. The phylogenetic tree was generated by MEGA 6.0 software with 35 full-length foxtail millet CIPK protein sequences. The NJ method was applied with 1,000 bootstrap replicates. The foxtail millet CIPK genes were divided into four subgroups (I-IV). Exon-intron structure analysis was performed via GSDS. The lengths of the exons and introns of each SiCIPK gene are shown proportionally. The black boxes represent exons, and the black lines represent introns.

The SiCIPK genes were mapped to all nine foxtail millet chromosomes. However, *SiCIPK35* was located in scaffold 47 because of a lack of complete chromosomal information. As shown in Fig 1, the SiCIPK genes are distributed across the 9 chromosomes, and similar to that which occurs for other CIPK family members, the SiCIPK genes are unevenly distributed across the genome [26, 39]. Among all the chromosomes, chromosome 5 contains the most (7) SiCIPK genes (20%), while chromosomes 2 and 3 each contain 6 genes (~17%), and at least one gene is located on chromosome 6 (~2.9%). On the basis of their phylogeny and gene structure, the 35 CIPK family members were divided into two clades with four subgroups (subgroup I to subgroup IV) (Fig 2A). Among the subgroups, subgroup I contains 4 members; the largest one, subgroup II, comprises 16 members. Subgroups III and IV contain 11 and 4 members, respectively. Exon/intron organization analysis further revealed that the SiCIPK genes can be clearly divided into an intron-poor clade (< 3 introns per gene, subgroups I and II) and an intron-rich clade (> 10 introns per gene, subgroups III and IV). In subgroups I and II, only *SiCIPK11* harbours two introns. In contrast, *SiCIPK2*, *-4*, *-6*, *-7*, *-13*, *-21*, *-22*, *-23*, *-25*, *-28* and -*32* harbour one intron each, and the other members are intronless (*SiCIPK10*, *-12*, *-14*, *-15*, *-18*, *-19*, *-24* and -*31*). In subgroups III and IV, with the exception of *SiCIPK29* and -*30*, the genes contain 20 introns, and the other members vary in intron number from 11 to 14 (Fig 2B).

### Conserved motif and evolution analysis of SiCIPK

**Fig 3:**
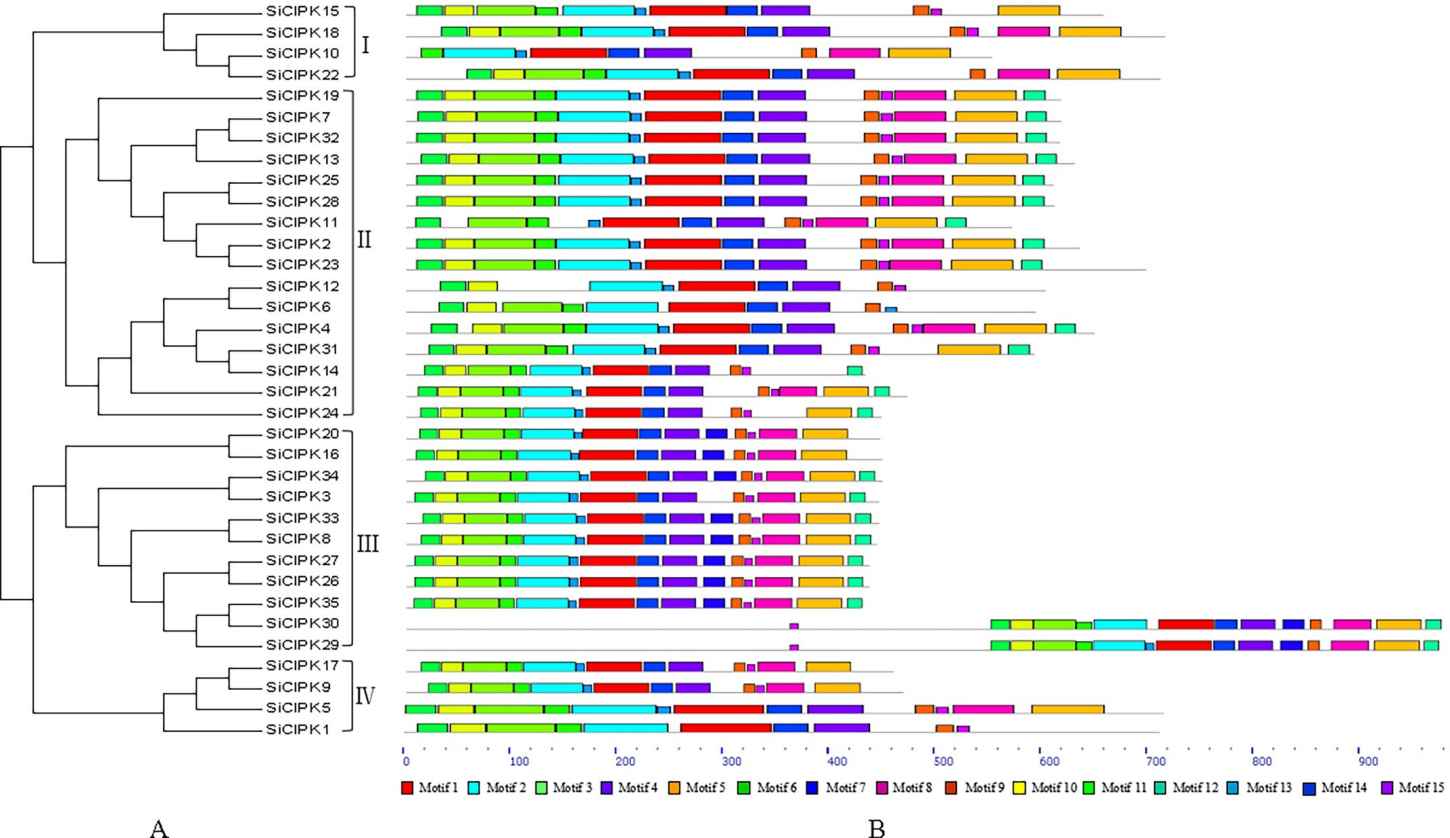
Conserved motifs of foxtail millet CIPK proteins according to their phylogenetic relationships. I, II, III and IV indicate the classification of foxtail millet CIPKs according to their phylogenetic relationships. All motifs were identified by MEME with the full-length amino acid sequences of the 35 CIPKs in foxtail millet. The different motifs are highlighted with different coloured boxes. The lengths of the motifs of each SiCIPK protein are displayed proportionally.

**Fig 4:**
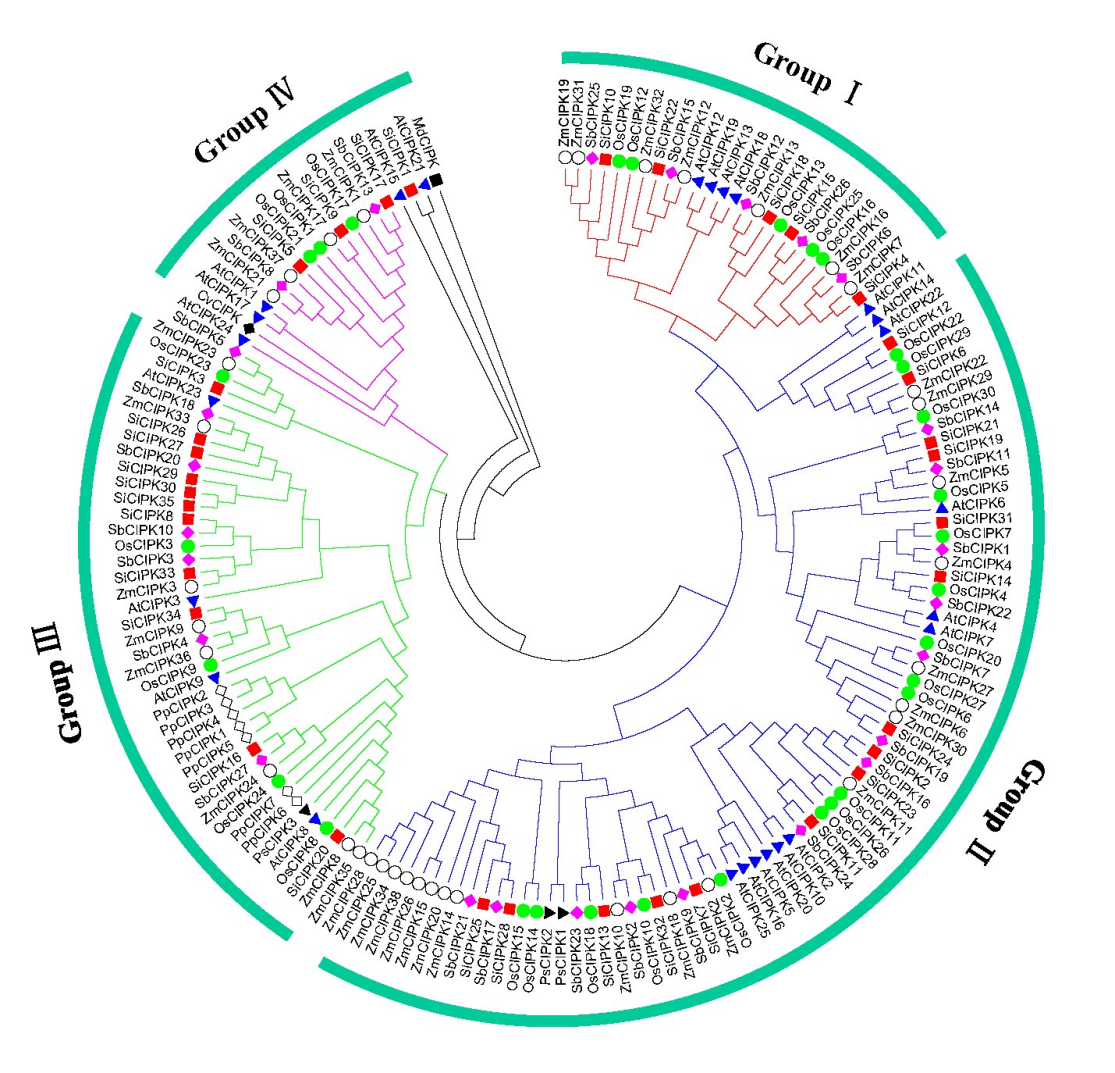
Evolutionary analysis of plant CIPK proteins. One hundred sixty-seven full-length CIPK protein sequences from *Arabidopsis*, rice, maize, sorghum, *Picea*, moss, green algae, and fungi were used to construct the phylogenetic tree via MEGA in conjunction with the NJ method. The subfamilies (groups I, II, III and IV) are highlighted with different colours, and the CIPK proteins in foxtail millet are marked by red squares.

To better understand the structural characteristics of the SiCIPK genes and provide clues to their functions, the conserved motifs of the SiCIPKs were analysed. We found 15 main conserved motifs, with all SiCIPK proteins having four core motifs: motifs 1, 4, 7 and 9 (Fig 3B). According to PROSITE, motifs 1, 2 and 3 are annotated as protein kinase domains. Motif 9 harbours the core NAF (Pfam No. PF03822) residues and is annotated as the NAF domain. The kinase domain and the NAF domain are the two major domains typical of CIPK proteins [4]. The NAF domain is necessary for binding between CBL and CIPK proteins, and formation of a stable complex of CBL and CIPK proteins is a prerequisite for the CBL-CIPK network to participate in the regulation of plant abiotic stress responses [5]. Analysis of the conserved motifs demonstrated the conserved structure of the members of the SiCIPK gene family (Fig 3 and S2 Table), which is consistent with the conserved nature of CIPK family members in other plant species [47, 48]. To determine the origin and evolution of SiCIPKs, we constructed a phylogenetic tree of foxtail millet and other species (*Arabidopsis*, rice, maize, sorghum, moss, *Picea*, green algae and fungi) via the software ClustalW 2.0 and MEGA 6.0 on the basis of full-length protein sequences. The analysis revealed that all CIPKs in the nine species could be divided into two clades and four subclades (I to IV) (Fig 4), which is consistent with the evolutionary analysis of SiCIPK in foxtail millet (Fig 2). The CIPKs in these plants evolved into their present states through continuous expansion of four subgroups, which is also consistent with the general consensus concerning the evolution and development of plant CIPKs [49, 50]. In addition, we found that the angiosperm (rice, *Arabidopsis*, maize, sorghum, and foxtail millet) and gymnosperm (*Picea*) CIPK proteins among the nine plant species could be divided into intron-poor and intron-rich clades (Fig 4). These results revealed that the intron-poor/rich clades of CIPKs may have originated before the divergence of gymnosperms and angiosperms approximately 300 Myr ago [51].

### Cis-regulatory elements and expression profile analysis of SiCIPKs in response to stress

**Fig 5:**
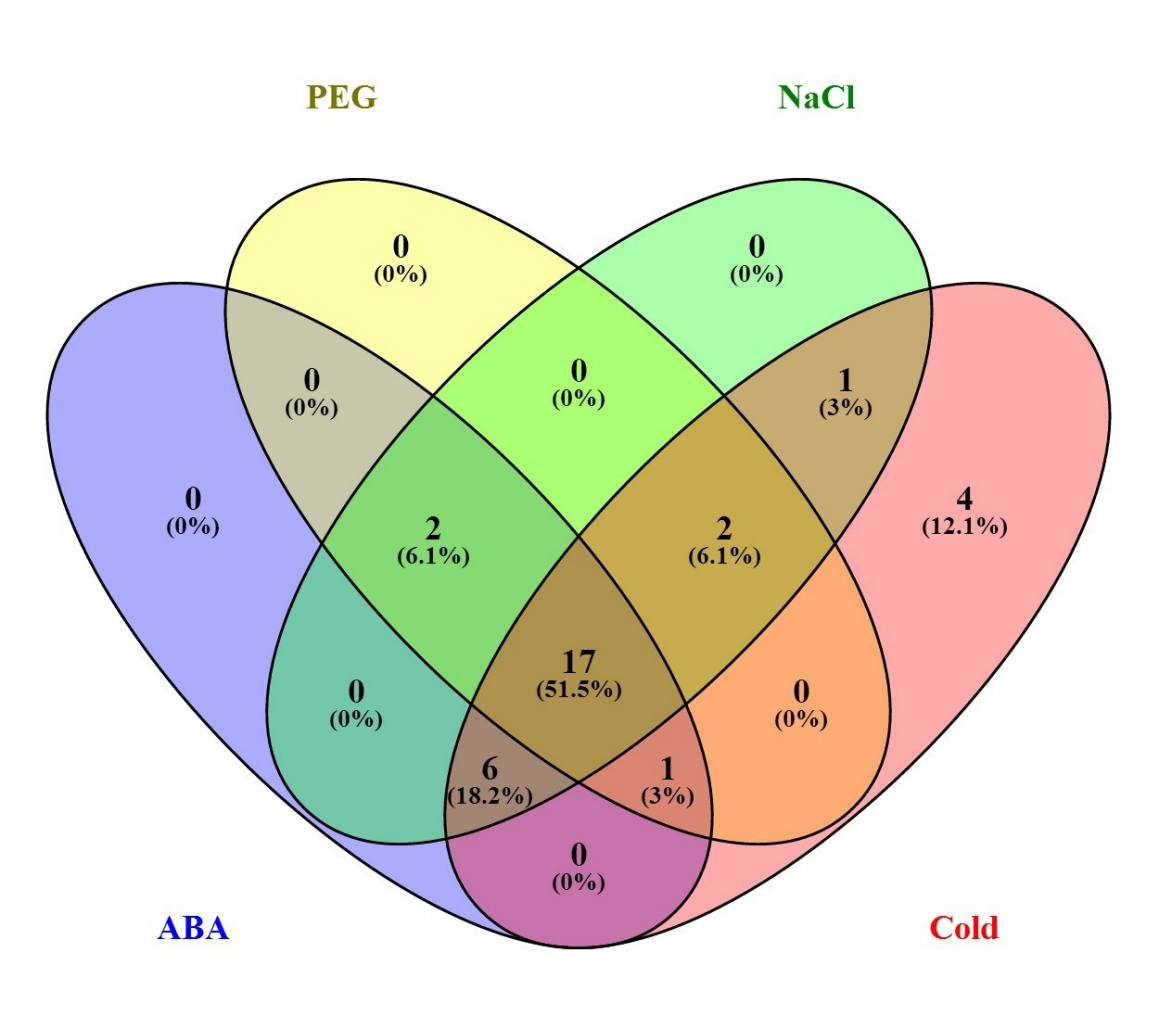
Venn diagram of SiCIPK genes whose expression is upregulated under stress treatments according to real-time PCR. The different treatments are highlighted with different coloured ellipses. The numbers represent the number of genes upregulated by each stress, and the numbers in brackets indicate the percentage of genes whose expression was induced among the total genes analysed.

**Fig 6:**
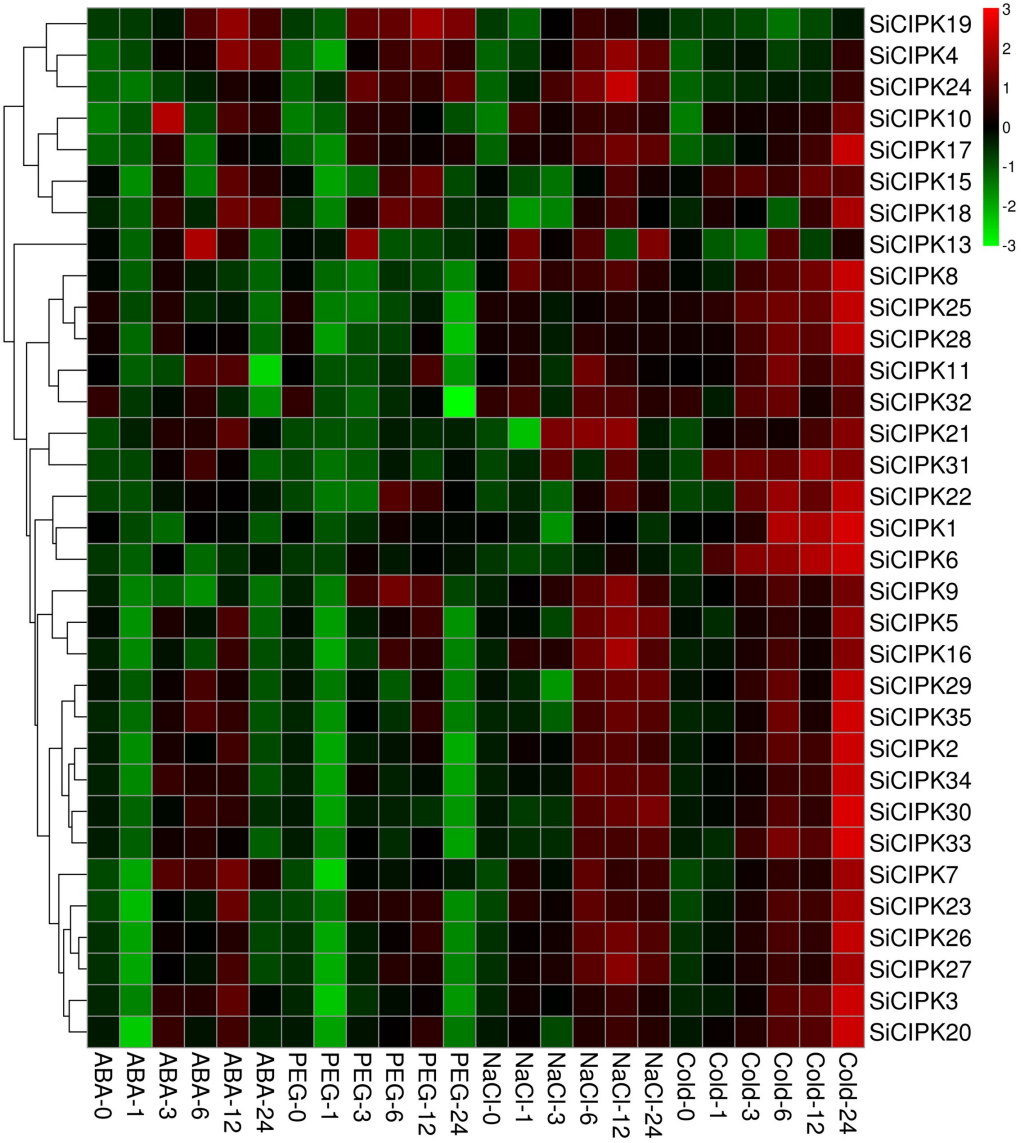
Heat map showing the expression profiles of SiCIPK genes in response to stress treatments. The relative expression was determined by qRT-PCR. The genes were hierarchically clustered on the basis of average Pearson distances. The scale bar in the upper right represents relative fluorescence intensity values that were log2 based.

Functional analysis of many identified CIPK family genes has demonstrated their important role in response to abiotic stress in plants [52, 53]. To determine the possible responses of SiCIPKs to abiotic stress, we examined the promoter regions of SiCIPKs for typical stress-responsive elements. We found a large number of stress-responsive elements within these promoter regions, including ABA-responsive elements (ABREs), MYB-binding sites (MBSs) involved in drought inducibility, defence and stress-responsive elements (TC-rich repeats) and low-temperature-responsive (LTR) elements (S3 Table). Additional stress-responsive cis-elements within SiCIPK gene promoter regions were found, including C-repeat/DRE, motif IIb, WUN-motif, CE3, fungal elicitor (Box W1), auxin (TGA-element) and endosperm expression (GCN4 motif and Skn-1 motif) elements, as well as those essential for anaerobic induction (ARE) (S4 Table). To verify the results of the cis-element analysis and further reveal the responses of SiCIPK genes to different stresses, the expression of all putative SiCIPK genes was measured via real-time PCR after PEG, salt, cold or ABA treatment. Among all the SiCIPKs, two (*SiCIPK12* and -*14*) were not detected because of their low expression. However, the expression of all the other 33 SiCIPK genes was upregulated in response to at least one stress; specifically, the expression of 32, 28, 33 and 33 genes was upregulated under ABA, PEG, cold and salt treatments, respectively (S5 Table). Our results clearly suggest that different SiCIPK genes exhibit different responses to stress (Figs 5 and 6). Furthermore, the transcript levels of some SiCIPK genes did not change obviously under certain treatments (e.g., *SiCIPK1*, -*25* and *-32* under ABA treatment; *SiCIPK1*, -*8*, -*25*, -*28*, -*30*, and -*32* under PEG treatment; and *SiCIPK1* under cold treatment). The expression of these genes varied little, and their level fluctuated around that of the controls (S6 Table). The expression of some genes was induced mainly by one or two stresses. For example, the expression of *SiCIPK1*, -*25*, -*28* and -*32* was strongly induced by only cold stress, and that of *SiCIPK8* was induced by cold and salt stress but not by ABA or PEG (S5 Table). These experimental data indicate that the CIPK family members in foxtail millet play important roles in stress responses.

### SiCIPK genes are involved in numerous common regulatory systems or cross-talk triggered by different stresses

To further investigate the responses of SiCIPKs to different stresses, we considered SiCIPKs to be stress-induced genes when their expression levels were more than twofold those of the controls for at least one time point. Among the 33 SiCIPK genes detected, the expression of 22, 26, 28 and 31 was induced by PEG, ABA, salt and cold treatments, respectively (Fig 5). We also found some SiCIPK genes to be responsive to multiple stresses. Among the 22 PEG-inducible genes, the expression of 20, 20, and 21 was also induced by ABA, cold and salt, respectively, and among the 28 salt-inducible genes, the expression of 25, 21, and 26 was also induced by ABA, PEG and cold, respectively. Similarly, among the ABA- and cold-inducible genes, the expression of a large number was induced by the other three treatments. Remarkably, the expression of 17 genes was induced by all four stress treatments (PEG, salt, cold and ABA) evaluated in this study (Fig 5). These results indicate that SiCIPK genes may be involved in numerous common regulatory systems or cross-talk triggered by different stresses. As many abiotic stresses lead to dehydration of plant cells and osmosis imbalance at the cellular level [54], the expression patterns of some stress-related genes in plants may overlap after exposure to various abiotic stresses such as drought, salt and cold as well as ABA. This phenomenon suggests that ABA and these stress signals share some elements in their signalling pathways and that these elements interact with each other to achieve a new cellular equilibrium [55, 56]. Our RT-PCR results also support this inference.

## Discussion

CIPKs are highly conserved and widely present in the plant kingdom. CIPK family members have been identified and characterized in many plant species [26, 39, 47, 48, 57, 58], and a large number of CIPK genes have been reported to be involved in the response to complex environmental stimuli in higher plants [59]. Although the foxtail millet genome has been sequenced and released [35, 38], there are very few reports of CIPKs in foxtail millet, especially in terms of responses to abiotic stress. In this study, 35 CIPK genes were identified in the foxtail millet genome and were mapped onto the nine foxtail millet chromosomes. Like CIPK family members in other plant species [4], SiCIPKs contain a conserved N-terminal catalytic kinase domain, a C-terminal regulatory domain and highly conserved NAF domain (S1 Fig).

Phylogenetic analysis divided the 35 CIPK family members of foxtail millet into two clades, including four subclades: I, II, III and IV (Fig 2A). One clade contains subclades I and II, and the other clade contains subclades III and IV. In addition, exon/intron organization analysis confirmed this classification. All SiCIPK genes were clearly divided into two distinct groups: an intron-poor group and an intron-rich group. The intron-poor group (< 3 introns per gene) included subgroups I and II, and the intron-rich group (> 10 introns per gene) included subgroups III and IV (Fig 2B). Gene structure analysis of the SiCIPKs supported the phylogenetic groupings of the SiCIPK family, and the intron-rich/poor pattern of the CIPK family in foxtail millet is similar to that in other species [60, 61]. Subsequent multiple sequence alignment analysis of CIPKs from nine representative plant species also revealed that all CIPKs could be divided into two groups (intron-poor and intron-rich groups) and four subgroups (I to IV) (Fig 4). This finding further confirmed the results of our multiple sequence alignment, conserved motif, and phylogenetic and organization analyses of SiCIPKs in foxtail millet (Figs 2 and 3). Moreover, phylogenetic analysis of the CIPKs from the nine species supported the amplification models of the plant CIPK gene family originating before the genome duplication within the four subgroups [49, 50, 60]. The divergence of angiosperms and gymnosperms occurred approximately 300 Myr ago [51], and we found many intron-poor and intron-rich CIPK genes in both angiosperms (rice, *Arabidopsis*, maize, sorghum, and foxtail millet) and gymnosperms (*Picea*) (Fig 4). This phenomenon revealed that the intron-poor/rich clade grouping may have originated before the separation of gymnosperms and angiosperms. These data indicated that intron gain and loss events have played important roles in the evolution of the CIPK family. Evolutionary analysis revealed many homologous gene pairs in foxtail millet and sorghum, maize, and rice, indicating a close relationship among these crop plant species, which is consistent with the current understanding of plant evolutionary history. In addition, the detection of homologous CIPK gene pairs with high sequence similarity from different species suggested that these genes may have similar functions in adaptation or evolution. There are many reports of highly homologous genes with similar molecular and biological functions in plants. For example, *MdCIPK6L*, *MdSOS2*, *ZmCIPK16*, and *SiCIPK24*, which are orthologous to *AtCIPK24*/*AtSOS2*, reportedly increase plant salt tolerance, with functions similar to those of *AtSOS2* [9–12]. *AtCIPK8*, an orthologue of *MeCIPK8*, is involved in regulating the low-affinity phase of the primary nitrate response [21]. *AtCIPK21*, which is highly similar to *MeCIPK21*, has a positive role in both osmotic and salt stress responses [62]. This evidence suggests possible roles for SiCIPKs and provides clues for future functional studies of SiCIPK genes in response to stress.

In general, the presence of a cis-element indicates that a gene may be involved in the response to the corresponding signal [63]. Abiotic stresses such as drought, salinity, low temperature, heat and wounding can lead to an increase in ABA in plant cells, and it is well known that ABA plays an important role in response to abiotic stress in plants [64–66]. Our cis-element analysis revealed that many stress-responsive cis-elements, such as ABRE, MBS, TC-rich repeats, LTR, C-repeat/DRE, motif IIb, and WUN-motif elements, are widely distributed within the promoter regions of SiCIPK members (S3 Table). Further stress expression profile analysis revealed that the expression of 33 SiCIPK genes was induced by at least one stress. The expression patterns and characteristics of these 33 genes after exposure to stress were diverse, with the expression of a few genes being induced by one stress (*SiCIPK1*, -*25*, -*28* and -*32* were strongly induced by cold) and the expression of most genes being induced by multiple stresses. Compared with those of the controls, some gene expression levels under a specific stress treatment did not change obviously, while the expression levels of some genes did change considerably (S6 Table, Figs 5 and 6). Furthermore, we found that the expression of 17 SiCIPK genes was simultaneously induced by the four stresses applied, indicating that almost half of the SiCIPK genes are involved in common regulatory systems or cross-talk triggered by different stresses. In addition, the expression of most of the SiCIPK genes was strongly induced under salt and cold treatments, but that of only a few SiCIPK genes was strongly induced under ABA and PEG treatments (Fig 6), indicating that foxtail millet seedlings are more sensitive to low-temperature and salt stresses than to ABA and PEG stresses. Thus, when foxtail millet seedlings are subjected to various adverse conditions, they will preferentially perceive and initiate responses to low temperature and salt. These results are consistent with the characteristics of foxtail millet, which is highly resistant to both salt and low-temperature stresses [36]. In addition, some hormone response elements were found in the promoter regions of all SiCIPK members, including GARE-motif, AuxRR-core, CGTCA-motif, TGACG-motif, ERE, TGA-element, TGA-box, TCA-element, SARE, P-box, TATC-box, ABRE, and motif IIb elements (S4 Table). Pathogen infection usually leads to increases in salicylic acid (SA), jasmonic acid (JA) and ethylene (ET) levels in cells, and these hormones are thought to play major roles in biotic stress responses [67]. The large number of plant hormone response elements within the SiCIPK promoter regions further suggested that SiCIPKs might play important roles in plant biotic stress responses. As a crop species that prefers short days and warm climates, foxtail millet is sensitive to photoperiod [68], and the discovery of many light-responsive elements suggests that SiCIPKs likely play very important roles in response to light. In short, the large variety of cis-elements widely distributed within the SiCIPK genes and the results of the expression profile analysis indicated that foxtail millet CIPK family members are likely involved in many biological processes, including both abiotic and biotic stress responses and hormone signalling during plant growth and development. The results of this experiment not only verified the predictions for stress-related cis-elements but also further confirmed that SiCIPK genes are involved in biological responses to stress and greatly contribute to the ability of foxtail millet to cope with adverse conditions. Although the functions of these SiCIPKs must still be analysed in detail, our work provided clues for future research on the functions of SiCIPK genes.

**Fig 7:**
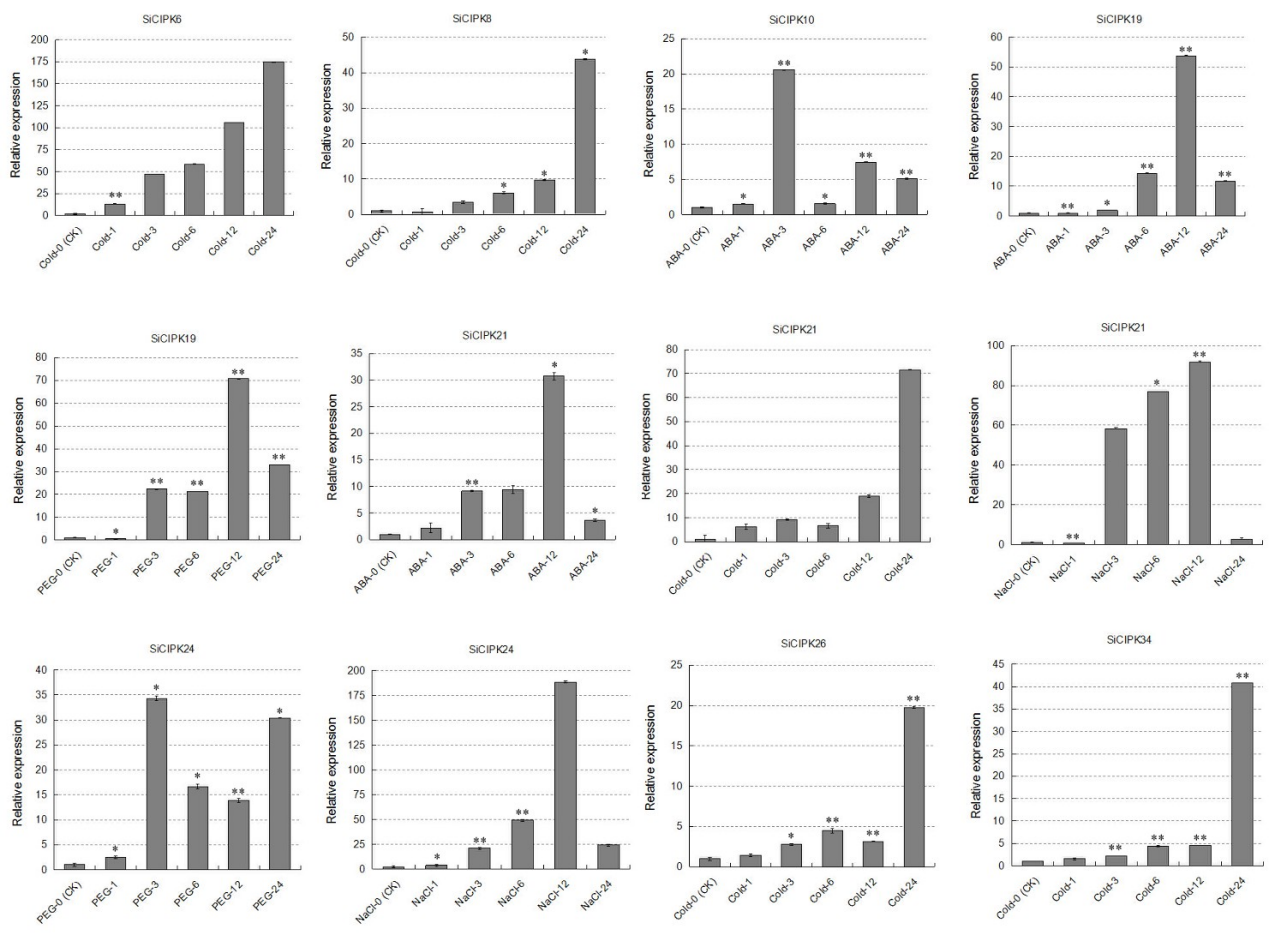
SiCIPKs whose expression is strongly induced by stress treatments. The relative expression levels of SiCIPKs were quantitatively calculated according to the 2^-ΔΔCt^ method. Gene expression was measured at six time points (0 h, 1 h, 3 h, 6 h, 12 h and 24 h). The Y-axis indicates the relative expression level, and the error bars represent the standard deviations calculated on the basis of three technical replicates for each biological duplicate. The asterisks indicate significant differences between the treatment and control according to Student's t test (**p < 0.01 and *p < 0.05).

We also noticed that the long N-terminal sequences of *SiCIPK29* and *SiCIPK30* render them the two longest *SiCIPK* genes (Figs 2 and 3). Sequence comparison revealed that *SiCIPK29* and *SiCIPK30* are highly homologous, with 81.36% sequence identity. Moreover, functional domain analysis revealed that, compared with other SiCIPKs, these proteins contain two copies of the N-terminal functional domain. Thus, their long sequence appears to be due to repetition of the N-terminal kinase domain, and we speculate that these two long genes may have evolved into their current states via replication of the N-terminal sequence. Regardless, no notable differences were found between them and the other SiCIPK genes with respect to cis-elements and expression under stress. We speculate that these genes may have additional functions in other signalling pathways. Remarkably, the expression of seven genes (*SiCIPK6*, *-8*, *-10*, *-19*, *-21*, *-24* and -*34*) was highly upregulated and was higher (≥ 20-fold) in the plants under stress than in the untreated control plants (Fig 7). Analysis of these highly induced expression profiles during stress treatments revealed several types of different expression profiles. In one type, which included *SiCIPK6*, *-8*, *-21*, *-26*, and *-34* under cold treatment, gene induction was rapid and transient in response to stress treatment, reaching a maximum at 24 h. In the second type (including *SiCIPK10*, *-19*, and *-21* under ABA treatment; *SiCIPK21* and *-24* under salt treatment; and *SiCIPK19* under PEG treatment), gene expression was induced after stress, peaked at 12 h (*SiCIPK10* under ABA treatment peaked at 3 h), and then decreased. In the third group, which contained only *SiCIPK24* under PEG treatment, gene expression was induced after stress, peaked at 3 h, decreased from 3 to 6 h, and then increased again at 24 h. These types represent the typical expression profiles of SiCIPK genes whose expression is induced under stress conditions. Together, these results indicate that members of the SiCIPK gene family exhibit stimulus-specific and time-dependent responses. It has been reported that the products of stress-inducible genes can be classified into two groups. The first group consists of functional proteins or proteins that probably directly protect against environmental stresses. The second group consists of regulatory proteins involved in further regulation of signal transduction and in the expression of genes that probably function in stress responses [69, 70]. Notably, SiCIPK genes belong to the second group, and they may be involved in further signal transduction and downstream gene expression in response to stress. Stress-inducible genes have been used to improve the stress tolerance of plants by gene transfer [71, 72]. It is important to analyse the functions of stress-inducible SiCIPK genes, not only to understand the molecular mechanisms of stress tolerance and the responses of higher plants but also to improve the stress tolerance of crops by genetic manipulation.

In summary, using sequence alignment methods, we identified 35 CIPK genes from the graminaceous C_4_ crop species foxtail millet. The members of the SiCIPK family have similar characteristic parameters and share high sequence identity with other plant CIPK genes according to protein parameter prediction, functional domain analysis, sequence alignment, and evolutionary analysis. Promoter cis-element analysis revealed that a large number of cis-elements related to stresses, hormones, light, and other growth and development processes were found in the promoter regions of the SiCIPK gene family members. qRT-PCR validation experiments revealed that the expression of 33 SiCIPK genes was induced by different stresses and that the expression of 17 SiCIPK genes was induced by four stresses simultaneously. Moreover, we found that foxtail millet seedlings are more sensitive to low-temperature and salt stresses than to ABA and PEG stresses. We also found that the expression of seven SiCIPK genes was highly induced by different stresses (S2 Fig). These results show that SiCIPKs exhibit the basic characteristics of CIPK family members and play important roles in response to abiotic stresses. It has been reported that intracellular Ca^2+^ concentrations temporarily fluctuate when plants are exposed to drought, salt, low temperature and ABA. As a Ca^2+^ sensor, CBL first perceives this change and interacts with its downstream CIPK to form a CBL/CIPK complex. The CBL/CIPK complex then phosphorylates downstream target proteins, relays various dynamic calcium signals and regulates related physiological processes. This activity causes a series of intracellular biochemical reactions, which enable plants to resist or adapt to various stresses [5]. The CBL/CIPK network is very complex. One CBL can interact with multiple CIPKs, and one CIPK can interact with multiple CBLs [23]. Accordingly, our future research will focus on elucidating the molecular mechanism of the interaction between SiCBLs and the highly induced SiCIPKs. Further identification of key components of the CBL/CIPK signalling pathway and analysis of related functions will allow a better understanding of how foxtail millet responds to abiotic stresses via the CBL/CIPK signalling system.

## Conclusions

In this study, 35 SiCIPK genes were identified in foxtail millet, a C_4_ Gramineae crop species known for its outstanding stress tolerance. Our systematic analysis revealed that SiCIPKs have basic characteristics similar to those of CIPK family members identified from other plant species, and we found that SiCIPK genes play important roles in stress responses. The results of this study increase the number of known CIPK members in plants and provide an experimental basis for further elucidating the functions and mechanisms of abiotic stress responses mediated by CIPKs in foxtail millet.

## Supporting information

S1 Fig Alignment of SiCIPK proteins. The red box indicates the kinase domain of SiCIPKs, and the blue box indicates the NAF domain of SiCIPKs.
(TIF)

S2 Fig Experimental steps and main conclusions. (TIF)

S1 Table SiCIPK gene primers used for RT-qPCR. (XLSX)

S2 Table Conserved motif analysis of SiCIPK family proteins. (XLSX)

S3 Table Stress-responsive cis-element analysis in the promoter regions of SiCIPK genes. (XLSX)

S4 Table Promoter analysis of the foxtail millet SiCIPK gene family members. (XLSX)

S5 Table Expression patterns of the SiCIPK gene family members under stress. (XLSX)

S6 Table RT-qPCR data for SiCIPK genes under stress. (XLS)

## References

[1] Zhu JK. Salt and drought stress signal transduction in plants. Annual Review of Plant Biology. 2002; 53: 247–273. 10.1146/annurev.arplant.53.091401.143329 12221975 PMC3128348

[2] Yu QY, An LJ, Li WL. The CBL-CIPK network mediates different signaling pathways in plants. Plant Cell Reports. 2014; 33(2): 203–214. 10.1007/s00299-013-1507-1 24097244

[3] Hu W, Xia ZQ, Yan Y, Ding ZH, Tie WW, Wang LZ, et al Genome-wide gene phylogeny of CIPK family in cassava and expression analysis of partial drought-induced genes. Frontiers in Plant Science. 2015; 6: 914 10.3389/fpls.2015.00914 26579161 PMC4626571

[4] Shi J, Kim KN, Ritz O, Albrecht V, Gupta R, Harter K, et al Novel protein pinases associated with calcineurin B-Like calcium sensors in Arabidopsis. Plant Cell. 1999; 11: 2393–2405. 10.1105/tpc.11.12.2393 10590166 PMC144142

[5] Albrecht V, Ritz O, Linder S, Harter K, Kudla J. The NAF domain defines a novel protein-protein interaction module conserved in Ca^2+^-regulated kinases. Embo Journal. 2001; 20: 1051–1063. 10.1093/emboj/20.5.1051 11230129 PMC145464

[6] Liu J, Ishitani M, Halfter U, Kim CS, Zhu JK. The Arabidopsis thaliana SOS2 gene encodes a protein kinase that is required for salt tolerance. Proc Natl Acad Sci. 2000; 97: 3730–3734. 10.1073/pnas.060034197 10725382 PMC16308

[7] Halfter U, Ishitani M, Zhu JK. The Arabidopsis SOS2 protein kinase physically interacts with and is activated by the calcium-binding protein SOS3. Proc Natl Acad Sci. 2000; 97: 3735–3740. 10.1073/pnas.040577697 10725350 PMC16309

[8] Qiu QS, Guo Y, Dietrich MA, Schumaker KS, Zhu JK. Regulation of SOS1, A plasma membrane Na^+^/H^+^ exchanger in Arabidopsis thaliana, by SOS2 and SOS3. Proc Natl Acad Sci. 2002; 99: 8436–8441. 10.1073/pnas.122224699 12034882 PMC123085

[9] Wang RK, Li LL, Cao ZH, Zhao Q, Li M, Zhang LY, et al Molecular cloning and functional characterization of a novel apple MdCIPK6L gene reveals its involvement in multiple abiotic stress tolerance in transgenic plants. Plant Molecular Biology. 2012; 79: 123–135. 10.1007/s11103-012-9899-9 22382993

[10] Hu DG, Li M, Luo H, Dong QL, Yao YX, You CX, et al Molecular cloning and functional characterization of MdSOS2 reveals its involvement in salt tolerance in apple callus and Arabidopsis. Plant Cell Rep. 2012; 31: 713–722. 10.1007/s00299-011-1189-5 22108717

[11] Zhao JF, Sun ZF, Zheng J, Guo XY, Dong ZG, Huai JL, et al Cloning and characterization of a novel CBL-interacting protein kinase from maize. Plant Molecular Biology. 2009; 69: 661–674. 10.1007/s11103-008-9445-y 19105030

[12] Zhang YM, Linghu JJ, Wang D, Liu X, Yu AL, Li FT, et al Foxtail Millet CBL4 (SiCBL4) interacts with SiCIPK24, modulates plant salt stress tolerance. Plant Mol Biol Rep. 2017; 35: 634–646. 10.1007/s11105-017-1051-1

[13] Xu J, Li HD, Chen LQ, Wang Y, Liu LL, H L, et al A protein kinase, interacting with two calcineurin B-like proteins, regulates K^+^ transporter AKT1 in Arabidopsis. Cell. 2006; 125(7): 1347–1360. 10.1016/j.cell.2006.06.011 16814720

[14] Huang C, Ding S, Zhang H, Du H, An L. CIPK7 is involved in cold response by interacting with CBL1 in Arabidopsis thaliana. Plant Science. 2011; 181: 57–64. 10.1016/j.plantsci.2011.03.011 21600398

[15] Yang WQ, Kong ZS, Omo-Ikerodah E, Xu WY, Li Qun, Xue YB. Calcineurin B-like interacting protein kinase OsCIPK23 functions in pollination and drought stress responses in rice (Oryza sativa L.). Journal of Genetics & Genomics. 2008; 35: 531–543. 10.1016/S1673-8527(08)60073-9 18804072

[16] He L, Yang X, Wang L, Zhu L, Zhou T, Deng J, et al Molecular cloning and functional characterization of a novel cotton CBL-interacting protein kinase gene (GhCIPK6) reveals its involvement in multiple abiotic stress tolerance in transgenic plants. Biochemical & Biophysical Research Communications. 2013; 435: 209–215. 10.1016/j.bbrc.2013.04.080 23660187

[17] Luan S, Lan WZ, Lee SC, Salt DE, Williams L. Potassium nutrition, sodium toxicity, and calcium signalling: connections through the CBL-CIPK network. Current Opinion in Plant Biology. 2009; 12: 339–346. 10.1016/j.pbi.2009.05.003 19501014

[18] Weinl S, Kudla J. The CBL-CIPK Ca2^+^-decoding signalling network: function and perspectives. New Phytologist. 2009; 184: 517–528. 10.1111/j.1469-8137.2009.02938.x 19860013

[19] Gao P, Zhao PM, Wang J, Wang HY, Du XM, Wang GL, et al Co-expression and preferential interaction between two calcineurin B-like proteins and a CBL-interacting protein kinase from cotton. Plant Physiol Biochem. 2008; 46(10): 935–940. 10.1016/j.plaphy.2008.05.001 18573665

[20] Tripathi V, Parasuraman B, Laxmi A, Chattopadhyay D. CIPK6, a CBL-interacting protein kinase is required for development and salt tolerance in plants. Plant Journal. 2010; 58(5): 778–790. 10.1111/j.1365-313X.2009.03812.x 19187042

[21] Hu HC, Wang YY, Tsay YF. AtCIPK8, a CBL-interacting protein kinase, regulates the low-affinity phase of the primary nitrate response. Plant Journal. 2009; 57: 264–278. 10.1111/j.1365-313X.2008.03685.x 18798873

[22] D' Angelo C, Weinl S, Batistic O, Pandey GK, Cheong YH, Schültke S, et al Alternative complex formation of the Ca-regulated protein kinase CIPK1 controls abscisic acid-dependent and independent stress responses in Arabidopsis. Plant Journal. 2006; 48: 857–872. 10.1111/j.1365-313X.2006.02921.x 17092313

[23] Kolukisaoglu U, Weinl S, Blazevic D, Batistic O, Kudla J. Calcium sensors and their interacting protein kinases: Genomics of the Arabidopsis and rice CBL-CIPK signalling networks. Plant Physiol. 2004; 134: 43–58. 10.1104/pp.103.033068 14730064 PMC316286

[24] Poonam K, Sibaji S, Indu T, Akhilesh KY, Amita P, Sanjay K, et al Comprehensive structural, interaction and expression analysis of CBL and CIPK complement during abiotic stresses and development in rice. Cell Calcium. 2014; 56(2): 81–95. 10.1016/j.ceca.2014.05.003 24970010

[25] Sun T, Wang Y, Wang M, Li T, Zhou Y, Wang X, et al Identification and comprehensive analyses of the CBL and CIPK gene families in wheat (Triticum aestivum L.). Bmc Plant Biology. 2015; 15: 269 10.1186/s12870-015-0657-4 26537110 PMC4634908

[26] Li LB, Zhang YR, Liu KC, Ni ZF, Fang ZJ, Sun QX, et al Identification and bioinformatics analysis of SnRK2 and CIPK family genes in Sorghum. Agricultural Sciences in China. 2010; 9: 19–30. 10.1016/S1671-2927(09)60063-8

[27] Mo CY, Wan SM, Xia YQ, Ren N, Zhou Y, Jiang XY. Expression Patterns and Identified Protein-Protein Interactions Suggest That Cassava CBL-CIPK Signal Networks Function in Responses to Abiotic Stresses. Frontiers in Plant Science. 2018; 9: 269 10.3389/fpls.2018.00269 29552024 PMC5841119

[28] Cui XY, Du YT, Fu JD, Yu TF, Wang CT, Chen M, et al Wheat CBL-interacting protein kinase 23 positively regulates drought stress and ABA responses. BMC Plant Biol. 2018; 18: 93 10.1186/s12870-018-1306-5 29801463 PMC5970481

[29] Abdula SE, Lee HJ, Ryu H, Kang KK, Nou I, Sorrells ME, et al Overexpression of BrCIPK1 Gene Enhances Abiotic Stress Tolerance by Increasing Proline Biosynthesis in Rice. Plant Mol Biol Rep. 2016; 34(2): 501–511. 10.1007/s11105-015-0939-x

[30] Tai FJ, Yuan ZH, Li SP, Wang Q, Liu FY, Wang W. ZmCIPK8, a CBL-interacting protein kinase, regulates maize response to drought stress.Plant Cell Tiss Organ Cult. 2016; 124(3): 459–469. 10.1007/s11240-015-0906-0

[31] Pan WH, Shen JQ, Zheng ZZ, Yan X, Shou JX, Wang WX, et al Overexpression of the Tibetan Plateau annual wild barley (Hordeum spontaneum) HsCIPKs enhances rice tolerance to heavy metal toxicities and other abiotic stresses. Rice. 2018; 11: 51 10.1186/s12284-018-0242-1 30209684 PMC6135728

[32] Yan Y, He XY, Hu W, Liu GY, Wang P, He CZ, et al Functional analysis of MeCIPK23 and MeCBL1/9 in cassava defense response against Xanthomonas axonopodis pv. Manihotis. Plant Cell Rep. 2018; 37(6): 887–900. 10.1007/s00299-018-2276-7 29523964

[33] Sheng LX, Meng XY, Wang M, Zang S, Feng LG. Improvement in Submergence Tolerance of Cherry Through Regulation of Carbohydrate Metabolism and Plant Growth by PsERF and PsCIPK. Appl Biochem Biotechnol. 2018; 184(1): 63–79. 10.1007/s12010-017-2530-4 28608173

[34] Guo YL, Huang Y, Gao J, Pu YY, Wang N, Shen WY, et al CIPK9 is involved in seed oil regulation in Brassica napus L. and Arabidopsis thaliana (L.) Heynh. Biotechnol Biofuels. 2018; 11: 124 10.1186/s13068-018-1122-z 29743952 PMC5930439

[35] Zhang G, Liu X, Quan Z, Cheng S, Xu X, Pan S, et al Genome sequence of foxtail millet (Setaria italica) provides insights into grass evolution and biofuel potential. Nature Biotechnology. 2012; 30(6): 549–554. 10.1038/nbt.2195 22580950

[36] Muthamilarasan M, Prasad M. Advances in Setaria genomics for genetic improvement of cereals and bioenergy grasses. Theoretical & Applied Genetics. 2015; 128: 1–14. 10.1007/s00122-014-2399-3 25239219

[37] Muthamilarasan M, Theriappan P, Prasad M. Recent advances in crop genomics for ensuring food security. Current Science. 2013; 104: 155–158.

[38] Bennetzen JL, Schmutz J, Wang H, Percifield R, Hawkins J, Pontaroli AC, et al Reference genome sequence of the model plant Setaria. Nature Biotechnology. 2012; 30(6): 555–561. 10.1038/nbt.2196 22580951

[39] Tang J, Lin J, Li H, Li X, Yang Q, Cheng ZM, et al Characterization of CIPK family in Asian Pear (Pyrus bretschneideri Rehd) and co-expression analysis related to salt and osmotic stress responses. Frontiers in Plant Science. 2016; 7: 15026 10.3389/fpls.2016.01361 27656193 PMC5013074

[40] Bailey TL, Williams N, Misleh C, Li WW. MEME: discovering and analyzing DNA and protein sequence motifs. Nucleic Acids Res. 2006; 34(suppl-2): W369–73. 10.1093/nar/gkl198 16845028 PMC1538909

[41] Hu B, Jin JP, Guo AY, Zhang H, Luo JH, Gao G. GSDS 2.0: an upgraded gene feature visualization server. Bioinformatics. 2015; 31(8): 1296–1297. 10.1093/bioinformatics/btu817 25504850 PMC4393523

[42] Lescot M, Déhais P, Thijs G, Marchal K, Moreau Y, Van de Peer Y, et al PlantCARE, a database of plant cis-acting regulatory elements and a portal to tools for in silico analysis of promoter sequences. Nucleic Acids Research. 2002; 30(1): 325–327. 10.1093/nar/30.1.325 11752327 PMC99092

[43] Larkin MA, Blackshields G, Brown NP, Chenna R, McGettigan PA, McWilliam H, et al Clustal W and Clustal X version 2.0. Bioinformatics. 2007; 23: 2947–2948. 10.1093/bioinformatics/btm404 17846036

[44] Tamura K, Stecher G, Peterson D, Filipski A, Kumar S. MEGA6: molecular evolutionary genetics analysis version6.0. Mol Biol Evol. 2013; 30: 2725–2729. 10.1093/molbev/mst197 24132122 PMC3840312

[45] Xu Y, Hui L, Li X, Jing L, Wang Z, Yang Q, et al Systematic selection and validation of appropriate reference genes for gene expression studies by quantitative real-time PCR in pear. Acta Physiologiae Plantarum. 2015; 37: 1–16. 10.1007/s11738-015-1784-0

[46] Livak KJ, Schmittgen TD. Analysis of relative gene expression data using real-time quantitative PCR and the 2^-ΔΔCt^. Methods. 2001; 25(4): 402–408. 10.1006/meth.2001.1262 11846609

[47] Sun T, Wang Y, Wang M, Li T, Zhou Y, Wang X, et al Identification and comprehensive analyses of the CBL and CIPK gene families in wheat (Triticum aestivum L.). Bmc Plant Biology. 2015; 15: 269 10.1186/s12870-015-0657-4 26537110 PMC4634908

[48] Xi Y, Liu J, Dong C, Cheng Z. The CBL and CIPK gene family in grapevine (Vitis vinifera): genome-wide analysis and expression profiles in response to various abiotic stresses. Frontiers in Plant Science. 2017; 8: 978 10.3389/fpls.2017.00978 28649259 PMC5465270

[49] Adams KL, Wendel JF. Polyploidy and genome evolution in plants. Current Opinion in Plant Biology. 2005; 8: 135–141. 10.1016/j.pbi.2005.01.001 15752992

[50] Kleist TJ, Spencley AL, Luan S. Comparative phylogenomics of the CBL-CIPK calcium-decoding network in the moss Physcomitrella, Arabidopsis, and other green lineages. Frontiers in Plant Science. 2014; 5: 187 10.3389/fpls.2014.00187 24860579 PMC4030171

[51] Bowe LM, Coat G, Depamphilis CW. Phylogeny of seed plants based on all three genomic compartments: extant gymnosperms are monophyletic and Gnetales' closest relatives are conifers. Proc Natl Acad Sci. 2000; 97: 4092–4097. 10.1073/pnas.97.8.4092 10760278 PMC18159

[52] Xiang Y, Huang Y, Xiong L. Characterization of stress-responsive CIPK genes in rice for stress tolerance improvement. Plant Physiology. 2007;144:1416–1428. 10.1104/pp.107.101295 17535819 PMC1914128

[53] Chaves-Sanjuan A, Sanchez-Barrena MJ, Gonzalez-Rubio JM, Moreno M, Ragel P, Jimenez M, et al Structural basis of the regulatory mechanism of the plant CIPK family of protein kinases controlling ion homeostasis and abiotic stress. Proc Natl Acad Sci. 2014; 111: 4532–4541. 10.1073/pnas.1407610111 25288725 PMC4210280

[54] Mahajan S, Tuteja N. Cold, salinity and drought stresses: An overview. Archives of Biochemistry and Biophysics. 2005; 444(2): 139–158. 10.1016/j.abb.2005.10.018 16309626

[55] Thomashow MF. Plant cold acclimation: Freezing tolerance genes and regulatory mechanisms. Annurevplant Physiolplant Molbiol. 1999; 50: 571–599. 10.1146/annurev.arplant.50.1.571 15012220

[56] Shinozaki K, Yamaguchi-Shinozaki K. Molecular responses to dehydration and low temperature: differences and cross-talk between two stress signaling pathways. Curr opin plant Biol. 2000; 3: 217–223. 10.1016/S1369-5266(00)00067-4 10837265

[57] Chen XF, Gu ZM, Xin D, Hao L, Liu CJ, Huang J, et al Identification and characterization of putative CIPK genes in maize. Journal of Genetics and Genomics. 2011; 38: 77–87. 10.1016/j.jcg.2011.01.005 21356527

[58] Yu Y, Xia X, Yin W, Zhang H. Comparative genomic analysis of CIPK gene family in Arabidopsis and Populus. Plant Growth Regulation. 2007; 52: 101–110. 10.1007/s10725-007-9165-3

[59] Tang RJ, Zhao FG, Garcia VJ, Kleist TJ, Yang L, Zhang HX et al Tonoplast CBL-CIPK calcium signaling network regulates magnesium homeostasis in Arabidopsis. Proc Natl Acad. Sci. 2015;112:3134–3139. 10.1073/pnas.1420944112 25646412 PMC4364200

[60] Zhu K, Chen F, Liu J, Chen X, Hewezi T, Cheng ZM. Evolution of an intron-poor cluster of the CIPK gene family and expression in response to drought stress in soybean. Scientific Reports. 2016; 6: 28225 10.1038/srep28225 27311690 PMC4911590

[61] Zhang H, Yang B, Liu WZ, Li H, Wang L, Wang B, et al Identification and characterization of CBL and CIPK gene families in canola (Brassica napus L.). BMC Plant Biology. 2014; 14(1): 8–8. 10.1186/1471-2229-14-8 24397480 PMC3890537

[62] Pandey GK, Kanwar P, Singh A, Steinhorst L, Pandey A, Yadav AK, et al Calcineurin B-Like protein-interacting protein kinase CIPK21 regulates osmotic and salt stress responses in Arabidopsis. Plant Physiology. 2015; 169(1): 780–792. 10.1104/pp.15.00623 26198257 PMC4577403

[63] Narusaka Y, Nakashima K, Shinwari ZK, Sakuma Y, Furihata T, Abe H, et al Interaction between two cis-acting elements, ABRE and DRE, in ABA-dependent expression of Arabidopsis rd29A gene in response to dehydration and high-salinity stresses. Plant J. 2003; 34: 137–148. 10.1046/j.1365-313x.2003.01708.x 12694590

[64] Nakashima K, Yamaguchi-shinozaki K. ABA signaling in stress-response and seed development. Plant Cell Reports. 2013; 32: 959–970. 10.1007/s00299-013-1418-1 23535869

[65] Verma V, Ravindran P, Kumar PP. Plant hormone-mediated regulation of stress responses. BMC Plant Biology. 2016; 16: 86 10.1186/s12870-016-0771-y 27079791 PMC4831116

[66] Zhang J, Jia W, Yang J, Ismail A M. Role of ABA in integrating plant responses to drought and salt stresses. Field Crops Res. 2006; 97: 111–119. 10.1016/j.fcr.2005.08.018

[67] Bari R, Jones JD. Role of plant hormones in plant defence responses. Plant Molecular Biology. 2009; 69: 473–488. 10.1007/s11103-008-9435-0 19083153

[68] Jia XP, Bai JY, Fan BY, Zhang GN, Shi GA, Hou DY, et al Cloning and sequence analysis of a putative CCT-motif gene in ten foxtail millet cultivars. The Journal of Animal & Plant Sciences. 2016; 26: 1526–1532.

[69] Bray EA. Plant responses to water deficit. Trends Plant Sci. 1997; 2: 48–54. 10.1016/S1360-1385(97)82562-9

[70] Hasegawa PM, Bressan RA, Zhu JK, Bohnert HJ. Plant cellular and molecular responses to high salinity. Annu. Rev. Plant Physiol. Plant Mol. Biol. 2000; 51: 463–499. 10.1146/annurev.arplant.51.1.463 15012199

[71] Bajaj S, Targolli J, Liu LF, Ho THD, Wu R. Transgenic approaches to increase dehydration-stress tolerance in plants. Mol. Breed. 1999; 5: 493–503. 10.1023/A:1009660413133

[72] Holmberg N, Bu¨low L. Improving stress tolerance in plants by gene transfer. Trends Plant Sci. 1998; 3: 61–66. 10.1016/S1360-1385(97)01163-1

