# Supplementary figures and images for: Foxtail millet (Setaria italica (L.) P. Beauv) CIPKs are responsive to ABA and abiotic stresses
Source: PLoS One. 2019 Nov 12;14(11):e0225091. doi: 10.1371/journal.pone.0225091 (PMC6850536; doi:10.1371/journal.pone.0225091)

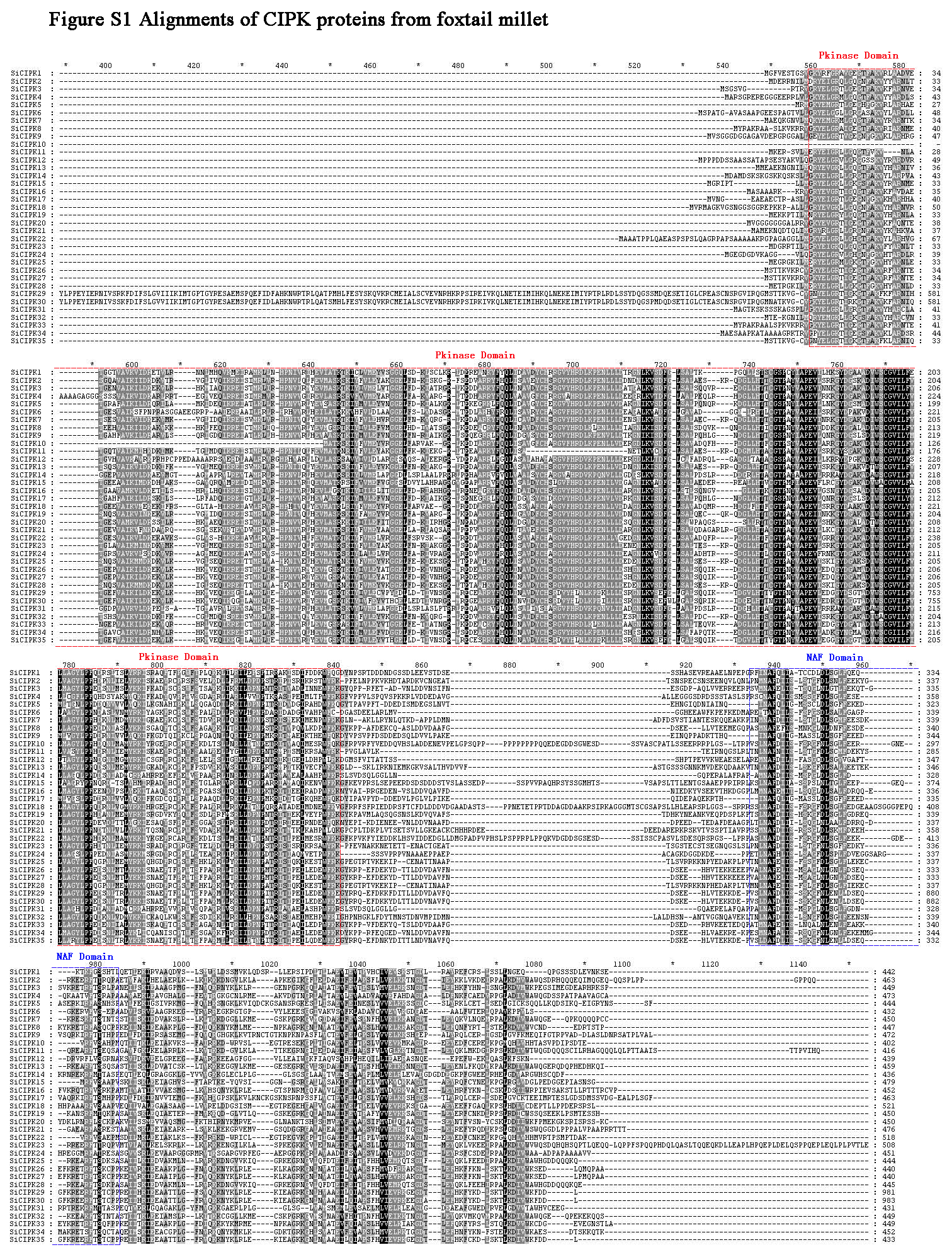

Supplement: S1 Fig — The red box indicates the kinase domain of SiCIPKs, and the blue box indicates the NAF domain of SiCIPKs. (TIF) [file pone.0225091.s001.tif]

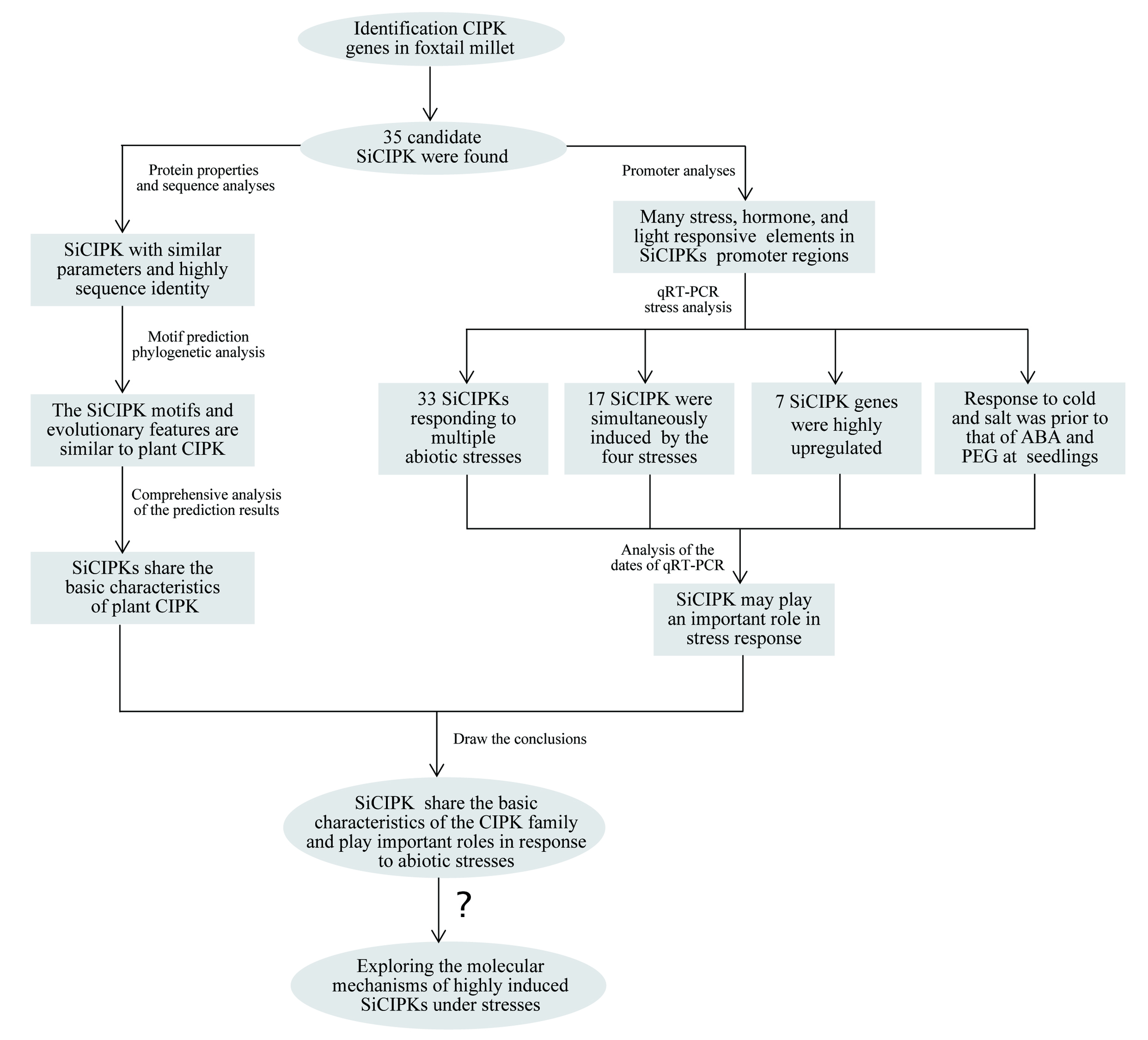

Supplement: S2 Fig — (TIF) [file pone.0225091.s002.tif]
